# Demonstration of a Fair Level of Agreement Between Escalation Scores Reported by Hospital Managers and Analysis of Stress-Related Hospital Metrics

**DOI:** 10.1177/2333392818819291

**Published:** 2019-03-15

**Authors:** Hugo C. van Woerden, Neil J. Walker, Vasiliki Kiparoglou, Yaling Yang

**Affiliations:** 1 Centre for Health Science, University of the Highlands and Islands, Inverness, UK; 2 Oxford Biomedical Research Centre, Churchill Hospital, Oxford, UK; 3 Nuffield Department of Primary Care Health Science, University of Oxford, Oxford, UK

**Keywords:** unscheduled care, escalation scores, hospital pressure, emergency admissions

## Abstract

**Background:**

The National Health System in Wales has developed a novel national electronic dashboard which reports a daily “escalation score,” reflecting management’s opinion of the pressure each hospital is facing, primarily due to unscheduled care. The aim of this study was to examine the possibility of replacing human scores with a quantitative model, based on the relationship between reported escalation scores and selected hospital metrics.

**Methods:**

Generalized linear mixed models were used to model the association between hospital metrics and escalation scores between October one year and October the next year utilizing hospital bed occupancy rate, ambulance hours lost waiting outside emergency departments, number of “boarded out” patients in the hospital, and the daily ratio of admissions to discharges in the hospital. These models were tested against a subsequent period (December unto May the following year), using three models: “general,” “hospital-specific,” and “group-specific.” The model generated by the initial time frame was tested against data from the subsequent time frame using weighted κ.

**Results:**

Across 16 hospitals, using 3343 escalation scores, the rates of agreement and weighted κ were: general model (48.8%; 0.16), hospital-specific model (45.0%; 0.25), and group-specific model (43.1%; 0.25). A 17th small hospital was excluded due to missing data.

**Conclusions:**

This is novel research as no similar studies were identified, although the topic is important as it addresses a major current health-care challenge. Automated scores can be derived which have the advantage of being derived objectively, avoiding human inter- and intraindividual variation. Prospective testing is recommended to assess potential service planning benefit.

## Background

The National Health System (NHS) is facing major pressure, with overfull hospitals struggling to cope with demand.^1^ This pressure has resulted in missed waiting time targets in emergency departments and difficulty in maintaining flow through hospitals, associated with occupancy rates close to 100%.^2^

This is important, as it is well established that inpatient overcrowding is associated with increased mortality and morbidity.^3^ The pressure experienced by hospitals has been widely reported in the media and it is recognized that new initiatives are required which provide real-time monitoring of hospital pressure and assessment of initiatives that manage to share the load of high occupancy rates across neighboring hospitals.^4^ To date, there has been very little modeling of the pressures that exist across different hospitals. There is the potential to use a number of pertinent metrics, which are routinely recorded, to monitor the pressure in different hospitals in an area centrally, based on demands on their capacity, in part related to fluctuations in the demand for unscheduled care related to emergency admissions. To a degree, these variations follow predictable short- and long-term trends.^5,6^ Part of the challenge in planning and responding to times of high pressure revolves around accurately assessing the level of unscheduled care demand and responding appropriately.^7,8^ Responses need to be coordinated across neighboring hospitals on a regional basis to ensure resilience of the health-care system as a whole.^9-11^

**Figure 1.**
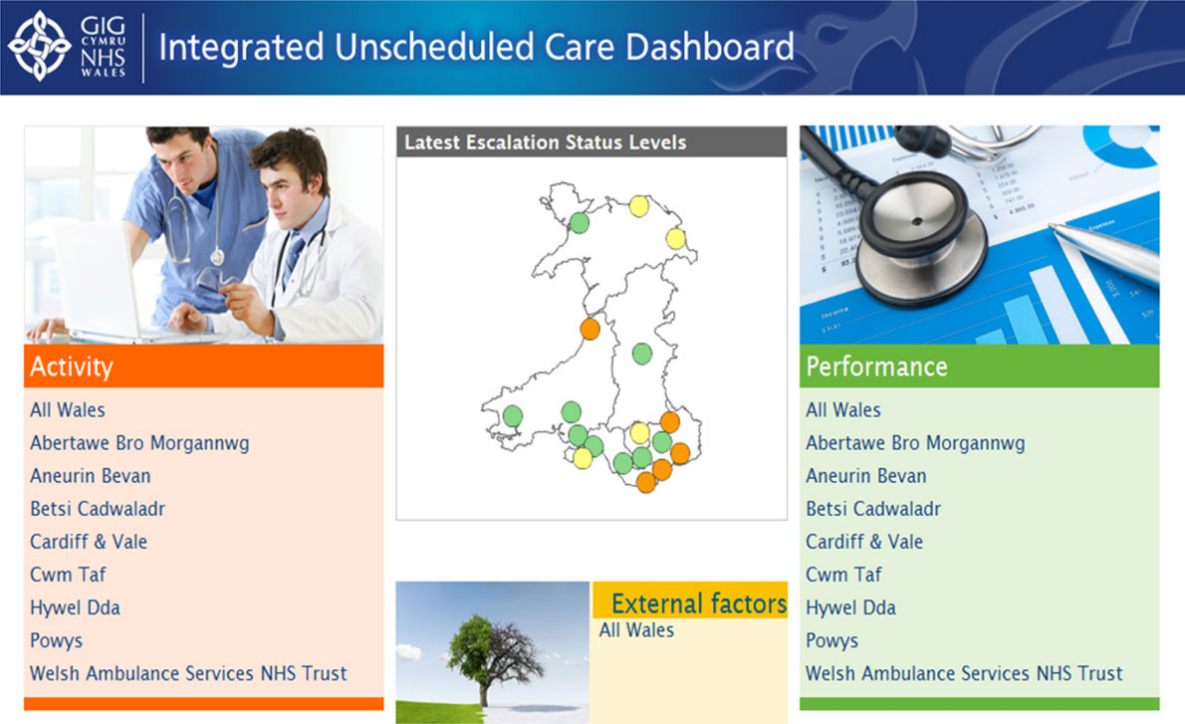
Capture of the Welsh dashboard, displaying real-time information on the pressure experienced and performance with respect to associate hospitals.

Quantifying pressure in hospitals can assist health-care planning, in terms of identifying where resources are most needed at any given time.^12^ Consequently, the NHS Wales operates a national online system whereby hospital managers report an escalation score for each hospital on a daily basis and, in some cases, when centrally requested, more than once a day. The escalation score represents the perceived pressure each hospital is experiencing. An example of one of the web pages from the national online Unscheduled Care Dashboard is shown in Figure 1.

Reported escalation scores, ranging from 1 to 4, are based on the following criteria:

Level 1: Steady state (hospital able to cope with current rate of admissions with available resources)

Level 2: Moderate pressure (admissions likely to exceed capacity)

Level 3: Severe pressure (admissions are exceeding capacity)

Level 4: Extreme pressure (admissions significantly exceeding capacity)

**Figure 2.**
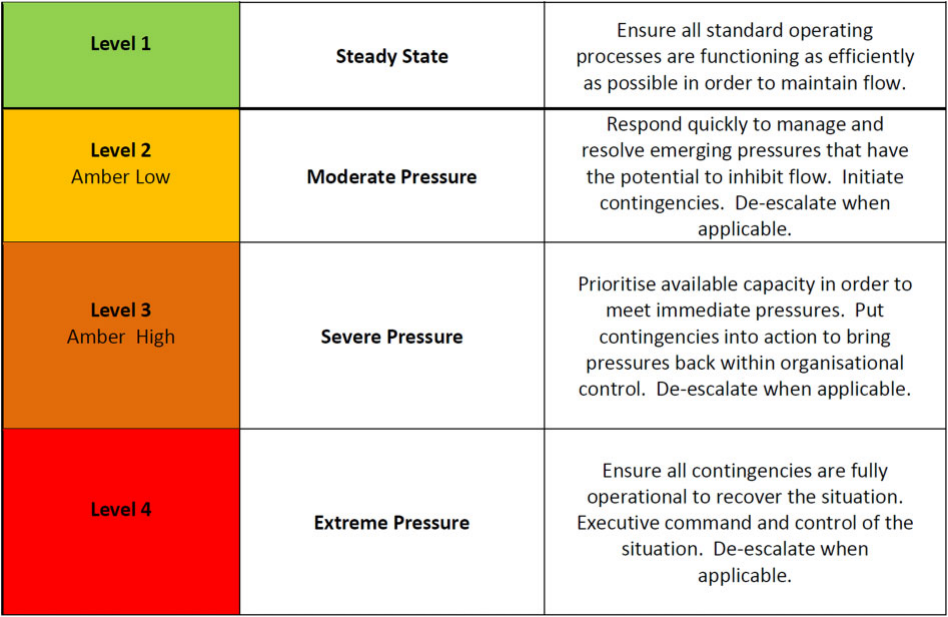
The definitions of the 4 states of escalation used by NHS Wales alongside the prescribed actions to be undertaken by hospitals categorized accordingly. NHS, National Health System.

Recommended actions to be taken by a hospital based on the reported score are listed in Figure 2.

The Welsh Government allows hospitals at level 4 to divert emergency admissions to other local hospitals to even out pressure across the Welsh health-care system. Further detail including the rationale behind the scores is provided by Piggott et al.^13^

Despite the guidelines by Piggott et al as to which factors should be taken into account when managers determine an escalation score, the system is intrinsically subjective. It is inevitable that hospital managers will sometimes assess pressure differently, both between and within hospitals. There is a risk that scores in the recent past may color assessment of the score for the present day and that scores in one hospital may influence scores reported elsewhere. A mechanism for generating pressure scores based on automated mathematical models, using routinely collected hospital metrics, is therefore attractive as it potentially avoids these different sources of subjectivity. National Health System Wales collects a range of metrics on a daily basis for their real-time dashboard which can assist in taking decisions across the health-care system.^14^

As well as collecting daily escalation scores from each hospital in Wales, the central electronic dashboard used by NHS Wales and the Welsh Government has a range of real-time data feeds, with some data fields automatically updated as frequently as every 10 minutes and other data fields updated manually on a daily basis. The presence of a range of variables in the national data set potentially provides an opportunity to derive an alternative, more objective assessment of pressure across the hospitals in Wales, which could replace escalation scores based on the opinion of local managers, with the associated risks of intra- and interindividual variation.

The aim of this study was to quantify the relationship between reported escalation scores and selected hospital metrics, whether or not it is feasible to implement in the NHS Wales system is not the focus of this article.

## Methods

Escalation scores from a full calendar year were obtained for the 17 hospitals in Wales that provide unscheduled care. Throughout this presentation, the hospitals in question are described with a number (1-17) assigned to preserve anonymity. The data set was provided by the NHS Wales Informatics Service, under the auspices of the Unscheduled Care Lead for Wales. No identifiable data were shared to ensure compliance with data protection regulations and to ensure that the research complied with the ethical standards for research set out in the Declaration of Helsinki.^15^

In order to facilitate analysis, a single escalation score was utilized per day for each hospital, although there were some days on which more than one score had been recorded. In such instances, the median value was used, with rounding to the nearest integer where necessary, and an upward rounding in the case of a decimal value of 0.5. Upward rounding reflected the fact that multiple scores in one day were likely to be triggered by rising rather than falling escalation scores as there was little incentive to submit a further escalation score on the same day unless pressure was believed to be rising.

The metrics available to model hospital pressure were bed occupancy rate for each hospital (as a percentage), the number of boarded out patients in the hospital, the ratio of admissions to discharges for the hospital during the preceding 24 hours, and the number of hours spent by ambulances waiting outside emergency departments to offload patients in the preceding 24 hours (the metric was only available at regional level, with Wales divided into north, mid, and south regions). The ratio of admissions to discharges had a correction applied, which involved adding a value of 1 to both the numerator and denominator to overcome the problem of intractable division where a value of 0 was present.

The above metrics were identified based on a clinical consensus as to potentially important explanatory variables in statistical models. To take seasonal effects into consideration, trigonometric transformations (sine and cosine) of the “annual angle” were calculated for each day of the year and the day of the week (Sunday to Saturday) and incorporated into the model, as both of these measures were identified as significant predictors of hospital pressure in work we have previously undertaken.^6^ The annual angle (referred to hereafter as θ) denotes the proportion of the year that has passed, expressed as an angle in radians, taking January 1 as the start of the year. This allows adjustment for seasonal variation in escalation scores, based on a sinusoidal wave pattern.^16^

By way of sensitivity analysis, 3 statistical models were fitted using a generalized linear mixed model procedure.^17^ In each of the 3 models, daily escalation score was modeled as an aggregated binomial process, where scores of 1, 2, 3, and 4 were mapped to values of 0, 1, 2, and 3, respectively, out of a putative maximum value of 3. Anticipated within-hospital temporal correlation with respect to reported escalation scores was modeled using first-order autoregressive error.^18^ This approach was adopted in order to reduce the impact of this potential source of nonindependence and reduce the chance of spurious findings.

Inference on explanatory variables was based on *F*-tests using adjusted degrees of freedom according to the method of Kenward and Roger.^19^ A similar approach was used in our analysis of pressure scores in a previous paper and a detailed description of the procedure is provided there in the study by Walker et al.^6^ All analyses were carried out in GenStat 18th edition.^20^

All 3 models started with the following explanatory variables: (i) sine of θ, (ii) cosine of θ, (iii) day of the week, (iv) log (% bed occupancy), (v) log (n admissions/n discharges), (vi) log (ambulance hours lost), and (vii) number of boarded out patients.

We were interested in assessing 3 secondary hypotheses, as a form of sensitivity analysis of our findings. Firstly, in relation to the question as to whether our findings would only apply very specifically in a Welsh context or whether, in principle, the data suggested that our model might be generalizable to other contexts. A general model has the appeal of being generalizable beyond the current context to other parts of the United Kingdom and possibly further afield, but pragmatically it may be expected that interhospital variability would limit the applicability of a global model of this kind. Secondly, we were interested in assessing whether or not explanatory variables were very specific to the context of a given hospital. Thirdly, we were interested in whether hospitals fell into natural clusters, where a model might work well for a particular type of hospital. We could have clustered hospitals in a variety of ways, but decided to choose the average level of pressure as a way of categorizing hospitals into low, medium, and high categories. This was because there was the possibility that the scoring system of 1 to 4 had ceiling or floor effects, which might make the score work in different ways across hospitals with different average levels of pressure.

Three distinct statistical models were therefore developed, which differed in terms of their generality. These can be described as follows: (i) general model for all hospitals, (ii) hospital-specific model, (iii) and group-specific model for hospitals experiencing similar pressure. The general model incorporated all of the available explanatory variables. The hospital-specific model was based on the same set of explanatory variables but included an additional intercept for each hospital, as well as all 2-way interactions between the hospital variable and other metrics. A third, intermediate approach (the group-specific model) was also developed based on placing hospitals into one of 3 groups according to whether the mean escalation score at that hospital was low, medium, or high, defined as follows: low group: mean escalation = 1 to <1.8; medium group: mean escalation = 1.8 to <2.5; and high group: mean = escalation 2.5 to 4. This group-based model was fitted in a similar way to the hospital-based model except that in this instance the intercepts and interactions were fitted at the group (rather than hospital) level.

In all 3 model specifications, a full model was fitted, and then a backward stepwise elimination procedure applied (using 5% significance as the inclusion/exclusion criteria) until only statistically significant effects remained. (Other methods of elimination were also assessed but did not markedly affect the model.) The coefficients from these models were then retained for the purposes of estimating escalation scores as described in our earlier article.^6^

### Validation Against Independent Data

A second data set was obtained consisting of the same metrics from the same hospitals over a 6-month period spanning the period December 1, 2014, to May 31, 2015, inclusive and thus subsequent to the original data set to which the above models were fitted. From this second data set, an estimated escalation score was calculated according to the following approach:

A linear predictor, $\hat{B}$, was calculated according to the following equation: $\hat{B}=\overset{k}{\underset{i=1}{\sum }}{\hat{\beta }}_{i}x_{i}\;$ where ${\hat{\beta }}_{1:k}$ are all the relevant coefficients as estimated (ie, $\hat{\beta }$ s for days of the week, $\hat{\beta }$ for sine of θ and for all other model variables) and $x_{1:k}$ denotes the data corresponding to the aforementioned coefficients (day of the week indicators, sine of θ, etc, for a given hospital on a given day). From $\hat{B}$, an estimated score ($\hat{E}$) can be calculated according to the function: $\hat{E}=\;1+\left(3\times \left[\left(\text{exp}\left(\hat{B}\right)\right)/\left(\left(1+\text{exp}\left(\hat{B}\right)\right)\right)\right]\right)$. This gives a score on a continuous scale between 1 and 4 inclusive. An estimate of escalation can then be generated by rounding to the nearest integer.

To evaluate the 3 approaches, the estimated escalation scores from this second data set were derived using the model coefficients in conjunction with observed daily metrics and then compared against reported escalation, based on the degree of agreement as assessed by (i) the percentage rate of agreement between estimated and reported scores and (ii) weighted Cohen κ statistic.^21^ The 2 methods were chosen, as we were unable to identify a definitive approach to assessment in this context.

The concordance tables summarized the frequency of agreement/disagreement between all combinations of estimated and observed categories (4 × 4 = 16 cells). The weighted κ statistic measures the degree of agreement between 2 categorical scales compared to what would be expected on the basis of chance (conditional on marginal totals with respect to the rows and columns of the concordance tables). The score takes any value between 0 and 1, with higher scores indicating a greater level of agreement. As implied, this statistic penalizes disagreement according to the degree of discrepancy between the observed and estimated scores.

## Results

Summary statistics are shown in Table 1 for available variables including the escalation scores, which showed some between-hospital variation, allowing for the fact that the score can only take values between 1 and 4. Notable variation was also seen between hospitals in relation to the daily number of boarded outpatients and (log of) ratio of hospital admissions/discharges.

**Table 1.** Summary Statistics for Key Metrics Available From 17 Welsh Hospitals.

		Escalation Score		Hospital Bed Occupancy (%)		Ambulance Hours Lost Outside Emergency Departments		Admissions		Discharges		Daily Number of Boarded Outpatients	
Hospital ID^a^	Group	n	Mean	n	Mean	n	Mean	n	Mean	n	Mean	n	Mean
1	2	296	2.30	234	97.09	244	22.12	210	16.49	211	7.04	236	3.75
2	2	348	2.13	231	97.57	280	21.02	218	41.81	218	36.20	231	16.96
3	2	354	2.46	155	96.48	284	21.25	108	70.35	108	4.93	158	12.31
4	3	327	2.65	321	99.23	259	41.31	279	52.23	278	47.77	322	10.28
5	1	322	1.80	318	96.54	268	40.79	282	52.74	282	58.22	322	29.40
6	2	349	1.99	224	91.57	281	21.19	212	21.85	212	18.58	225	5.63
7	3	298	2.69	296	97.81	237	21.63	247	43.26	247	39.03	297	19.79
8	1	340	1.57	328	95.16	282	40.77	294	73.43	294	60.23	336	18.78
9	3	283	2.77	279	99.35	228	41.03	237	121.06	236	117.97	282	26.33
10	1	353	1.75	293	93.35	284	21.21	251	47.29	248	5.56	294	4.41
11	3	355	2.98	355	97.81	283	40.78	274	45.06	274	44.09	355	2.29
12	3	355	2.98	355	97.90	283	40.78	274	82.47	274	85.15	355	28.39
13	2	347	1.89	347	97.73	282	20.81	255	31.22	255	21.33	347	10.46
14	2	291	2.23	176	97.95	237	37.91	138	38.50	134	4.07	176	30.97
15	2	336	2.28	84	96.18	269	37.61	33	78.18	34	39.71	84	7.20
16	2	258	1.85	1	91.25	240	38.83	0	-	0	-	1	4.00
17	1	204	1.38	197	97.61	165	42.28	163	25.46	162	18.33	199	0.00

^a^ Hospital ID is denoted with a numeric code to preserve anonymity.

Table 1 also highlights the fact that the metrics were not recorded comprehensively and the degree to which data were missing for some hospitals. Given that individual records were omitted from analysis if any of the metrics were missing, the number of observations for analysis was reduced from a maximum possible of 6205 (17 hospitals × 365 days) to 3343 (46.1% of total possible hospital days had at least 1 item of data missing). Hospital 6 was excluded from analysis, as records of some variables were completely absent.

Backward stepwise elimination yielded the following most parsimonious models:

1. General model for all hospitals: y=day+log(occupancy)+log(hours lost)+ log(admissions/discharges)+boarded out patients.
2. Hospital-specific model: y=cosine(θ)+log(hours lost)+log(admissions/discharges)+boarded out patients +group×sine(θ)+group×log(occupancy)+group×day.
3. Group-specific model: y=log(hours lost)+boarded out patients+hospital×sine(θ)+hospital×cosine(θ)+hospital×day+hospital×log(admissions/discharges).

Where an interaction was present in the final model, the constituent main effects were automatically retained.

Tables 2, 3, and 4 show rates of agreement/disagreement when the estimated escalation scores produced from the 3 respective modeling strategies were compared to observed scores using the second data set. Agreement was highest with respect to the first model (48.8%) and was 43.1% and 45.0% for the second and third models, respectively.

**Table 2.** Concordance Statistics for Estimated Versus Observed Scores (Concordance Table With Frequencies of Each Combination and Weighted κ) for the General Model for All Hospitals.^a^

		Observed			
		1	2	3	4
Estimated	1	1	0	0	0
	2	33	35	14	3
	3	257	484	1045	390
	4	0	10	127	174

^a^ n (%) agreement = 1255 (48.8%) of 2573; weighted κ = 0.16.

**Table 3.** Concordance Statistics for Estimated Versus Observed Scores (Concordance Table With Frequencies of Each Combination and Weighted κ) for Hospital-Specific Model.^a^

		Observed			
		1	2	3	4
Estimated	1	25	19	28	4
	2	105	166	218	32
	3	144	278	589	201
	4	17	66	351	330

^a^ n (%) agreement = 1110 (43.1%) of 2573; weighted κ = 0.25.

**Table 4.** Concordance Statistics for Estimated Versus Observed Scores (Concordance Table With Frequencies of Each Combination and Weighted κ) for Group-Specific Model in Which Hospitals Are Grouped According to Average Pressure Reported (Low, Medium, and High).^a^

		Observed			
		1	2	3	4
Estimated	1	18	12	16	1
	2	97	139	179	28
	3	168	340	693	230
	4	8	38	298	308

^a^ n (%) agreement = 1158 (45.0%) of 2573; weighted κ = 0.25.

The weighted κ statistics for the 3 models were 0.16, 0.25, and 0.25, respectively. The first model performed less well on this metric than the second and third models, which were indistinguishable. The level of agreement achieved by the latter 2 approaches would be classed as “fair” based on the widely used scale for κ values, that is, poor, fair, moderate, good, and very good.^22^

## Discussion

In this analysis, we have considered management’s subjective escalation scores when assessing the pressure in Welsh hospitals against a set of 3 statistical models using available hospital metrics to generate automated escalation scores using the same scale of 1 to 4. We believe that the research presented in this article is novel. A few hospitals have experimented with local assessment of the pressure in a hospital (personal correspondence), but we have not been able to identify published research on the topic.

All of the explanatory measures included were significant in some capacity across the respective models, although not universally so in every case. Where statistically significant, the coefficients for these metrics were exclusively positive, indicating that higher values of these metrics were associated with greater reported pressure.

Simple agreement rates favored the general model as a method for predicting reported escalation, but weighted κ favored those models that included a characteristic of the hospitals involved. The elevated rate of agreement seen in relation to the general model can in part be attributed to a high frequency of estimates of an escalation score at “level 3” (severe pressure), such that 2176 (84.6%) of 2573 estimates fell into this category, as compared to 1212 (47.1%) of 2573 and 1431 (55.6%) of 2573 for the hospital-specific model and the model for groups of similar pressure hospitals, respectively. “Severe pressure” was the most commonly reported category (1186/2573 = 46.1%) in the second data set; thus, it can be understood why a model which frequently estimates this level of escalation is more likely to garner a high level of “3 × 3” agreement. However, this will also lead to lower levels of agreement in other categories. Furthermore, a score that cannot distinguish well between different levels of pressure is likely to be of limited use.

The high, medium, and low categories in the group-specific model were chosen based on the mean level of pressure over the period October 2013 to October 2014. There are different ways in which hospitals could be categorized that may be pertinent to the question including hospital size (eg, number of beds) and geographical location, and an improvement may be achieved if a more relevant categorization is identified.

We recognize this possibility of intercorrelation between the 4 variables chosen for the initial model, but where two predictors are strongly correlated, it is unlikely that they will both be included in a parsimonious model, as one of them is likely to fall out of the model on the basis of a *P* value that is less significant than the threshold chosen for excluding variables, that is, *P* > .05.

The possibility should be considered that differences between human assessment and that based on the statistical models may have been due to inconsistency in escalation scores generated by human intuition. Subjective escalation scores are not an ideal comparator, but it is difficult to conceptualize an alternative without the collection of additional data. Subtle factors, based on human judgment, that were expressed within the guidelines for managers when estimating an escalation score were perhaps less well captured using our automatically generated algorithm, and this may have contributed to poorer relationships between escalation scores and our analysis of hospital metrics.

Objective estimates of pressure clearly address some of the issues associated with a more subjective approach as outlined in the “Introduction.” It would be instructive to explore whether further metrics additional to those considered in the current analysis could be incorporated to improve upon the presented models.^23^ Since a key issue for hospitals is flow through the system, related to difficulty in discharging patients who are medically fit for discharge, some measure(s) of surgical and medical turnover or length of stay in hospital (particularly length of stay beyond the point when patients are medically fit for discharge) may be useful additional information to include in these models. This is an important area of research, given the evidence that hospitals that are operating near to 100% of capacity have adverse effects on patient and staff outcomes.^24,25^

Despite the presence of missing data, which it is acknowledged may have affected the results, the analysis nonetheless included more than 3000 records. Data for given metrics tended to be missing in blocks, more so in the earlier part of the time frame considered here and would appear to be related to the robustness of data collecting systems in place. This being the case, it would be unlikely that data were missing in such a way as to skew the results.

It would be helpful to determine whether objective models can predict the pressure on hospitals several days in advance, allowing preemptive action to be undertaken, such as the cancelation of planned surgery and triggering support from other hospitals, so as to more rapidly restabilize the system as a whole. Given the autocorrelation observed in reported scores at individual hospitals over consecutive days, this may represent a profitable area for investigation.

## Conclusion

The Welsh unscheduled care dashboard is a novel and potentially very useful development in managing the pressure across hospitals across a wider geographical area. The current study has considered a number of automated statistical models that can form the basis of algorithms to calculate an objective pressure score. There is a need to assess whether scores derived from statistical models in this way result in more effective management of unscheduled care pressures at a national level.
